# Prospective cohort study evaluating efficacy and safety of efgartigimod in Chinese generalized myasthenia gravis patients

**DOI:** 10.3389/fneur.2024.1407418

**Published:** 2024-06-17

**Authors:** Pan Wang, Bo Zhang, Jian Yin, Jianying Xi, Ying Tan, Feng Gao, Fan Zeng, Ting Chang, Hao Zhou, Hui Liang, Zhongyan Zhao, Huan Yang, Chongbo Zhao, Shixiong Huang

**Affiliations:** ^1^Department of Neurology, Hainan General Hospital, Hainan Affiliated Hospital of Hainan Medical University, Haikou, China; ^2^Department of Neurology, Beijing Hospital, Beijing, China; ^3^Department of Neurology, Huashan Hospital, Fudan University, Shanghai, China; ^4^Department of Neurology, Peking Union Medical College Hospital, Beijing, China; ^5^Department of Neurology, Peking University First Hospital, Beijing, China; ^6^Department of Neurology and Center for Clinical Neuroscience, Daping Hospital, Army Medical University, Chongqing, China; ^7^Department of Neurology, Tangdu Hospital, The Fourth Military Medical University, Xi’an, China; ^8^Department of Neurology, Xiangya Hospital, Central South University, Changsha, China

**Keywords:** efgartigimod, generalized myasthenia gravis, myasthenia gravis activities of daily living, quantitative myasthenia gravis, named patient program

## Abstract

**Background:**

Despite the efficacy of efgartigimod demonstrated in ADAPT phase 3 trial, data specifically derived from Chinese participants are not available. Therefore, we aimed to evaluate the efficacy and safety of efgartigimod in Chinese patients with generalized myasthenia gravis (gMG).

**Methods:**

This is a prospective cohort study conducted in 8 hospitals across China. gMG patients received weekly intravenous infusions of efgartigimod (10 mg/kg) under a named patient program (NPP). The present study is an 8-week study, consisting of 4 consecutive doses of efgartigimod administered over 3 weeks (one cycle), followed by a 5-week follow-up period to assess the tolerability of efgartigimod’s therapeutic effects. The primary outcome was the mean change in MG activities of daily living (MG-ADL) total score from baseline to 4 weeks. MG-ADL responder was defined as a ≥ 2-point improvement that persisted for 4 weeks, starting by week 4. Safety evaluations encompassed the monitoring of adverse events (AE) and serious AE (SAE) throughout the study.

**Results:**

Between 5 July 2022 and 25 August 2023, a total of 14 gMG patients were included. The mean age was 57.7 years, with a mean MG-ADL score of 10.86 ± 3.32. At week 4, MG-ADL scores showed a mean reduction of 6 points, reaching a maximum decline of 13 points. Among the patients, 85.7% (12/14) achieved MG-ADL responder status after one cycle of treatment. The most significant reduction in quantitative MG (QMG) scores also occurred at week 4, with a mean decrease of 7 points. Notably, the improvements in MG-ADL and QMG scores persisted until week 8. During treatment and follow-up period, only two mild neck rashes occurred and resolved promptly. No infections or SAE were reported.

**Discussion:**

A single cycle of efgartigimod treatment demonstrates effectiveness and the tolerability through week 8, with no new safety signals observed in Chinese gMG patients.

## 1 Introduction

Myasthenia gravis (MG) is an autoimmune disorder characterized by localized or widespread manifestations of muscle weakness, with about 85% of patients having IgG autoantibodies against the nicotinic acetylcholine receptor (AChR) in skeletal muscle (1). Generalized MG (gMG) severely impairs functions like speech, swallowing, breathing and eye movements, affecting quality of life (1, 2). In China, MG prevalence is rising (3), with an incidence rate of 0.68 per 100,000 person-years and a mortality rate upon admission of 14.69 per thousand (4). Current treatment options for Chinese MG patients primarily consist of thymectomy, pyridostigmine, non-steroidal immunosuppressants (NSIST), glucocorticoids such as prednisolone or methylprednisolone, intravenous immunoglobulin and plasma exchange (5). Patients with gMG may benefit from the depletion of B cells (e.g., CD20 monoclonal antibody) (6). These treatments are tailored to individual patient requirements, focusing on symptom management and minimizing side effects (7). However, there are some limitations to these approaches, making it crucial to develop more effective strategies for patients.

Efgartigimod, an Fc fragment of human IgG1, targets and reduces pathogenic immunoglobulin G (IgG) antibodies by blocking their recycling via the neonatal Fc receptor (FcRn), critical in preventing IgG degradation (8, 9). Results from phase I and II trials demonstrate its efficacy in lowering IgG without impacting other immunoglobulins or albumin, offering potential in treating IgG-mediated autoimmune diseases (10). The ADAPT phase 3 trial highlighted the efficacy and tolerability of efgartigimod in gMG, demonstrating consistent benefits across various MG scales (11). However, the limited representation of Asians, particularly the absence of Chinese patients, underscores the need for additional assessment of efgartigimod within this population. Additionally, the efgartigimod was not approved in China when the study was initiated. To bridge this gap, we launched a named patient program (NPP).

## 2 Materials and methods

### 2.1 Study design

This prospective cohort study was conducted at 8 centers in China. Efgartigimod was not approved in China when the study commenced in July 2022. Therefore, it was provided for compassionate use under an NPP, allowing the use of unapproved drugs when no satisfactory alternatives were available. This program in China was conducted in strict adherence to ethical guidelines to safeguard patient safety. Eligible patients were administered weekly intravenous infusions of efgartigimod at a dosage of 10 mg/kg, while maintaining their existing MG medication regimen. For patients weighing ≥ 120 kg, a maximum dose of 1,200 mg per infusion was administered. The whole treatment comprised a single cycle, consisting of 4 consecutive doses administered over a period of 3 weeks, with scheduled weekly visits (V1–V4, week 0–3). This was followed by visits at 1 week (week 4) and 5 weeks (week 8) after the last dose (V5, V6; Supplementary Figure 1). Efgartigimod treatment and visits V1-5 were conducted at Hainan General Hospital, while the screening and V6 were completed at other centers. This study adhered to the principles outlined in the Declaration of Helsinki, and the ethics committees and institutional review boards of each participating hospital provided approval for the study protocol. Written informed consent was obtained from all participants. The study was registered with the Chinese Clinical Trial Registry (registration number: ChiCTR2200066880).

### 2.2 Participants

Enrolled Chinese patients in this study were 18 years or older and diagnosed with gMG. Patients were eligible if their diseases were classified as MG Foundation of America (MGFA) class II to IV, presenting with a MG activities of daily living (MG-ADL) score of 5 or higher (>50% non-ocular) and a positive AChR-Ab test. Moreover, they had received one or more stable treatments (defined as treatment remained unchanged for the past month) for gMG (limited to acetylcholinesterase inhibitors, steroids, and/or NSIST) before screening. Patients were excluded if they had (1) received treatment with rituximab or eculizumab within 6 months, thymectomy within 3 months, or IVIg or PE within 1 month prior to screening, (2) active hepatitis B, serological response for hepatitis C, low CD4 cell count for HIV, serum IgG levels below 6 g/L, or were pregnant.

### 2.3 Efficacy assessments

In assessing disease burden/severity, various MG-related scales such as the MG-ADL profile score (12), quantitative MG (QMG) (13), and MG composite scales (14) were utilized. The revised 15-item Myasthenia Gravis Quality of Life scale (MG-QoL15r) (15) and the EuroQoL 5-Dimensions 5-Levels (EQ-5D-5L) (16) were employed to assess patients’ self-perceived HR-QOL and the impact of MG on their daily functioning and overall well-being. The primary outcome of this study was the improvement in MG-ADL scores from baseline to the week 4 visit of the cycle (1 week post the fourth infusion) (11). The MG-ADL responder was defined as a persistent improvement of ≥2 points for a minimum of 4 consecutive weeks, with the first improvement occurring by week 4 (1 week after the last dose). Secondary outcomes included (1) reduction in QMG scores from baseline after 4 weeks of efgartigimod treatment and the proportion of QMG responders (defined as a persistent improvement of ≥3 points in total QMG score for at least 4 consecutive weeks, with the first improvement occurring by week 4) (11); (2) reduction in MG-QoL15r scores from baseline post 4 weeks of efgartigimod treatment; (3) improvement in EQ-5D-5L scores post 4 weeks of efgartigimod treatment; (4) percentage reduction in IgG levels in patients; and (5) the correlation between the percentage reduction in IgG levels and the percentage reduction in MG-ADL scores.

### 2.4 Safety assessments

Safety assessments encompassed monitoring the incidence of adverse events (AEs) and serious AEs (SAEs), as well as changes in laboratory examination-related parameters throughout the study. These examinations, carried out at each visit, included lymphocyte count, blood white blood cell (WBC) count, urine WBC count, neutrophil count, low-density lipoprotein cholesterol (LDL-C) level, total cholesterol (TC) level and albumin level.

### 2.5 Measurements of IgG and AChR-Ab

Patients’ IgG levels were measured using the turbidimetric inhibition immunoassay, while the AChR antibody levels were detected using radioimmunoassay (RSR limited, United Kingdom).

### 2.6 Statistical analysis

Continuous variables were initially assessed for normality, and those meeting normal distribution were expressed as mean ± standard deviation or mean ± standard error. Categorical variables were described as frequencies (%). The correlation between the percentage reduction in IgG and the percentage reduction in MG-ADL scores was analyzed using simple linear regression. The comparison of MG-ADL and QMG scores across different visits was analyzed using paired t-test. A *p*-value of less than 0.05 indicates statistical significance.

## 3 Results

### 3.1 Patient’s baseline characteristics

A total of 14 gMG patients completed 4 infusions and 5-week follow-up between July 5, 2022 and August 25, 2023. Patient baseline characteristics are shown in Supplementary Table 1. The mean age was 57.7 years, and the duration of gMG was 6.2 years. The majority of the patients (71.4%) were classified as MGFA class III, and more than half (8/14, 57.1%) had undergone thymectomy more than 1 year before enrollment. Four patients (28.6%) had no history of prior NSIST, steroid treatment or any other immunotherapy, except for pyridostigmine. Despite receiving ongoing systemic therapy for MG, their mean baseline MG-ADL and QMG scores were notably high at 10.86 ± 3.32 and 20.00 ± 5.02, respectively (Table 1).

**Table 1:** Baseline characteristics of the patients.

	Patients (*n* = 14)
Age (years, mean ± SD)	57.7 ± 13.3
**Sex, *n* (%)**	
Female	7 (50%)
Male	7 (50%)
Duration of gMG (years, mean ± SD)	6.2 ± 5.0
**MGFA, *n* (%)**	
III	10 (71.4%)
IV	4 (28.6%)
**Thymectomy, *n* (%)**	
Yes	8 (57.1%)
No	6 (42.9%)
Baseline MG-ADL (score, mean ± SD)	10.86 ± 3.32
Baseline QMG (score, mean ± SD)	20.00 ± 5.02
Baseline MG-QOL 15r (score, mean ± SD)	19.79 ± 5.89
**Prior treatment, *n* (%)**	
Only steroid	1 (7.1%)
Only NSIST	5 (35.7%)
Steroid and NSIST	4 (28.6%)
No steroid or NSIST	4 (28.6%)

gMG, generalized myasthenia gravis; NSIST, non-steroidal immunosuppressants; MG-ADL, myasthenia gravis activities of daily living; QMG, quantitative myasthenia gravis; MG-QOL 15r, 15-item Myasthenia Gravis Quality of Life Scale; SD, standard deviation; MGFA, myasthenia gravis foundation of America.

### 3.2 Efficacy of efgartigimod

**Figure 1:**
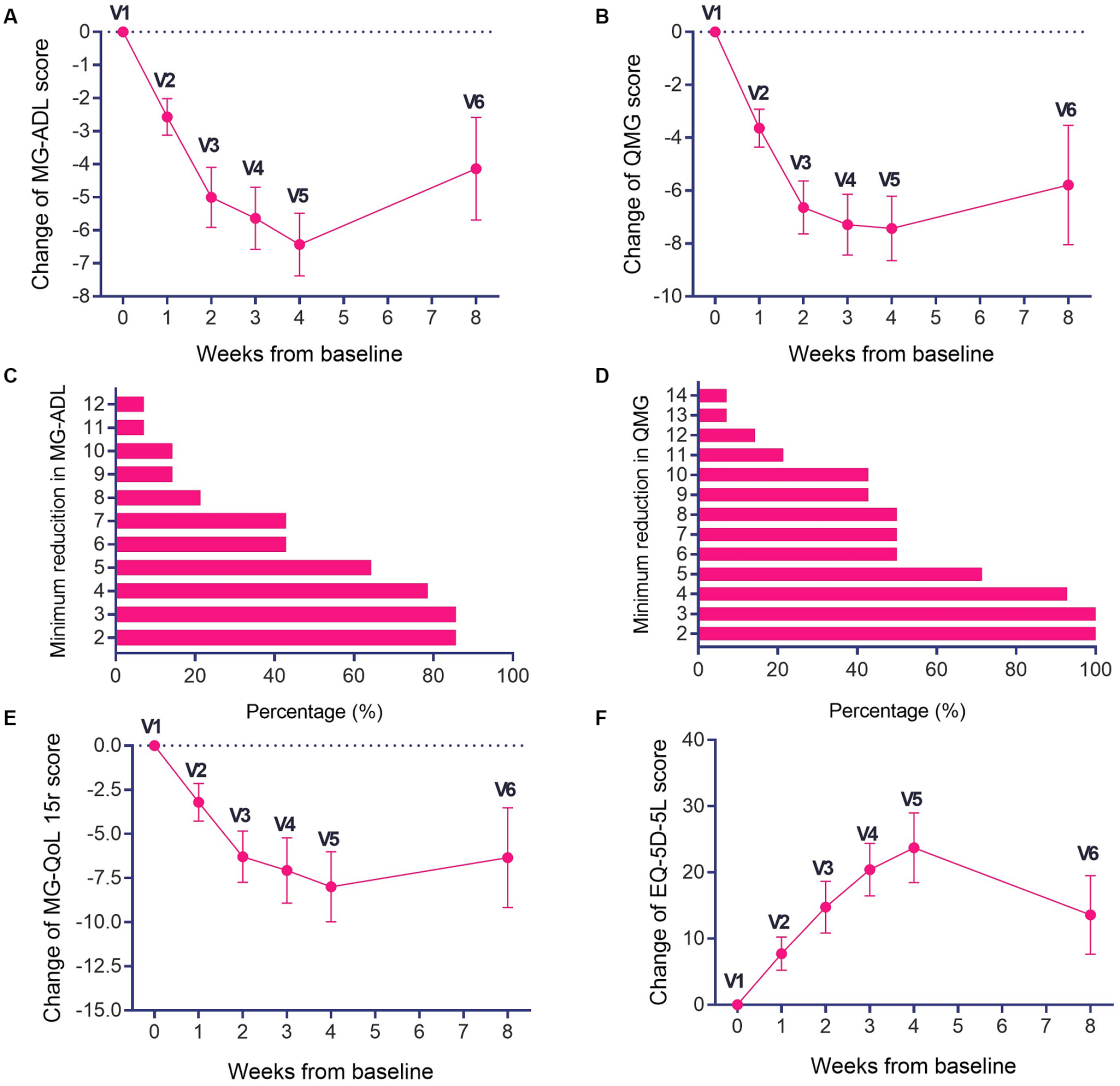
Changes in MG-ADL, QMG, MG-QoL15r scores and EQ-5D-5L scores during the treatment period and follow-up period. **(A)** Changes in MG-ADL and **(B)** QMG scores during the treatment and follow-up periods. The proportions of patients with different thresholds of **(C)** MG-ADL reduction and **(D)** QMG reduction in one treatment cycle. **(E)** Changes in MG-QoL15r and **(F)** EQ-5D-5L scores during the treatment period and follow-up period. ^*^p < 0.05, compared with V1.

After efgartigimod treatment, both MG-ADL and QMG scores decreased rapidly. Following a single cycle of treatment, significant improvements were observed in both MG-ADL (Figure 1A) and QMG scores (Figure 1B) at week 4. The most significant improvement in MG-ADL scores occurred during this period, with a mean decrease of 6 points (Figure 1A) and a maximum decrease of 13 points. Among the patients, 85.71% (12/14) were MG-ADL responders (Figure 1C), while those with a 3-point reduction in QMG scores reached 100% (Figure 1D). Of the 12 MG-ADL responders, 75% (9/12) experienced an onset of response by week 1, increasing to 100% by week 2. At week 8 (5 weeks after the last dose, V6), 78.6% (11/14) remained MG-ADL responders. The mean ADL scores at weeks 2, 3, 4, 8 exhibited significant decreases compared to the baseline (*p* < 0.05). The most significant reduction in QMG scores also occurred at week 4, with a mean decrease of 7 points (Figure 1B) and a maximum decrease of 17 points.

Patients exhibited a rapid decline in MG-QoL15r scores, achieving a maximum decrease of 25 points (Figure 1E), with the most significant improvement noted at week 4. Although the descent in MG-QoL15r scores slowed during the follow-up, there was still an obvious decrease compared to baseline (*p* < 0.05). It is evident that the MG-QoL15r scores showed a consistent downward trend during both the treatment and follow-up periods of efgartigimod (Figure 1E). Furthermore, the EQ-5D-5L scores showed improvements during the treatment period, with a maximum improvement of 60 points, and the mean peak improvement occurred at week 4, reaching 24 points (Figure 1F). Despite a slight deceleration in the trend of improvement in EQ-5D-5L scores during the follow-up, the improvement in EQ-5D-5L scores during both treatment and follow-up remained notably distinct compared to the baseline (*p* < 0.05; Figure 1F).

### 3.3 Safety

Only 2 mild AEs were reported, both manifesting as red rashes on the anterior neck, resulting in an AE incidence rate of 14.3% over the 8 weeks. No infections or SAE occurred throughout the course of the study. The changes in lymphocyte count, blood WBC count, LDL-C, urine WBC count, neutrophil count and TC were within normal limits (Supplementary Figure 2).

### 3.4 Changes in total IgG and AChR-Ab levels

**Figure 2:**
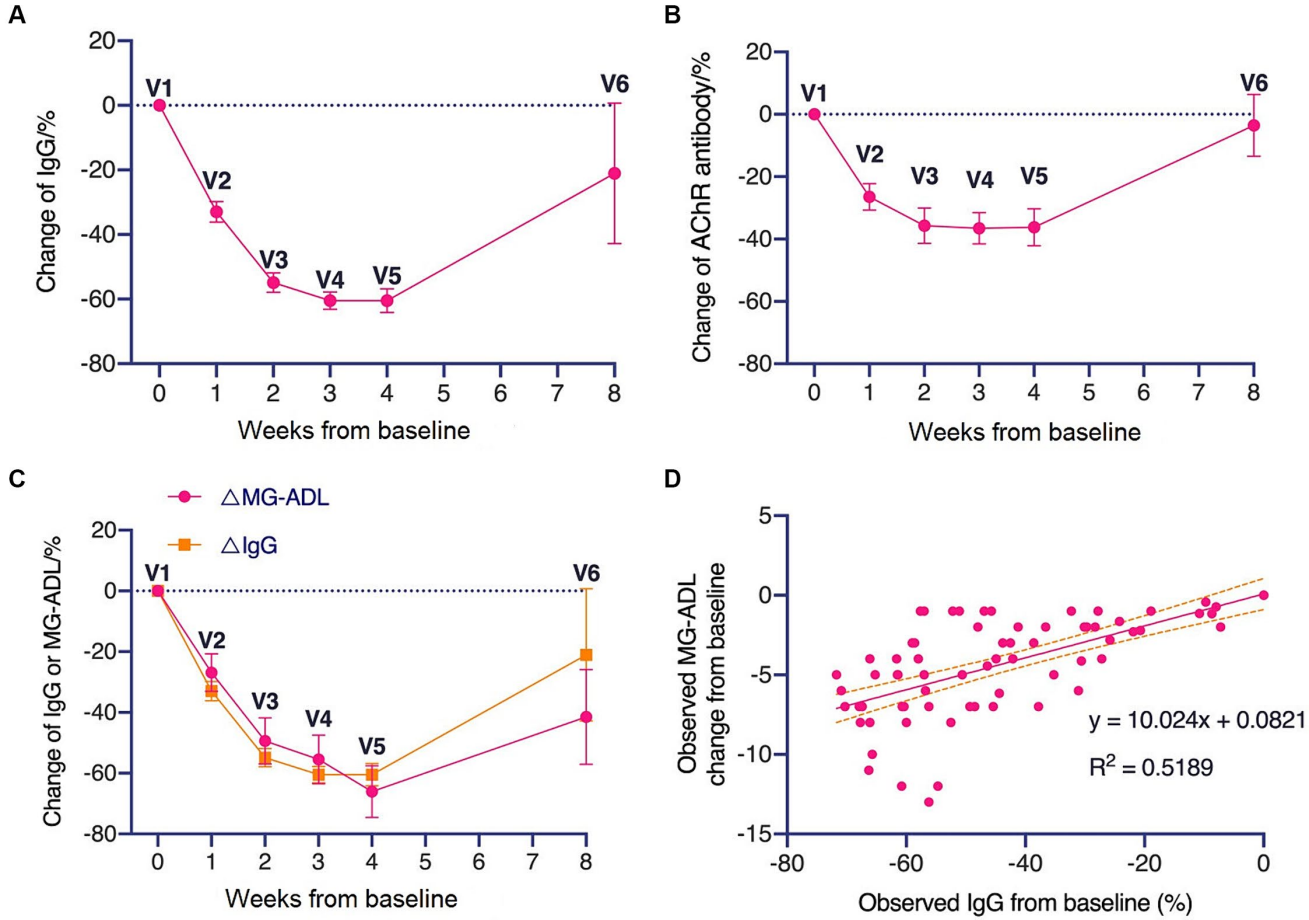
Changes in total IgG and AChR antibody levels during treatment period and follow-up period. **(A)** Changes in total IgG level and **(B)** AChR antibody level during treatment period and follow-up period. **(C)** Comparison of the trend of decreasing IgG levels with the trend of decreasing MG-ADL levels. **(D)** Linear correlation between changes in MG-ADL scores relative to baseline and changes in IgG levels relative to baseline.

The IgG levels and AChR-Ab levels of patients during the treatment period declined rapidly within 3–4 weeks (Figures 2A,B). A consistent trend between the decline in IgG levels and the decrease in MG-ADL scores was observed (Figure 2C). There was a linear correlation (R^2^ = 0.519, *p* < 0.0001) between the change in MG-ADL scores and the change in IgG levels from baseline (Figure 2D).

## 4 Discussion

This is the first study to investigate Chinese gMG patients treated with efgartigimod, and the results regarding efficacy and safety were consistent with those observed in the ADAPT study (11). Our study showed that a single treatment cycle of efgartigimod was effective, and its therapeutic effects seemed to last over a period of 5 weeks. Efgartigimod was well-tolerated in Chinese gMG patients with no new safety signals.

Based on the ADAPT subset analysis of Japanese population, only 37.5% (3/8) efgartigimod-treated patients achieved MG-ADL responder status during cycle 1 treatment in the overall population. However, the limited sample size in that analysis may have introduced bias. In our study, patients experienced rapid and significant improvements in MG-ADL, QMG scores and their overall QOL. Only 1 week after the last dose of efgartigimod, most patients exhibited improvements. Moreover, these improvements persisted even after a 5-week discontinuation of efgartigimod, indicating the tolerability of its therapeutic effects.

The patients in our study had a shorter time since diagnosis (over 6 years) compared to efgartigimod group in the ADAPT study (10 years). Our patients had limited treatment options, with 71.4% having received steroid and NSIST, in contrast to 81% in the ADAPT (11). Despite some differences in patient characteristics, the efficacy of efgartigimod in Chinese gMG patients remains comparable to that observed in the ADAPT study. After a single cycle of efgartigimod treatment, both the MG-ADL and QMG scores improved rapidly, with all patients demonstrating clinically meaningful improvement starting from the second week. In our study, 85.71% of the patients achieved MG-ADL responder status, compared with 68% in the ADAPT study, while the overall magnitude of change in MG-ADL and QMG scores aligned with observations from the ADAPT study (11). The subgroup analyses from the ADAPT trial have shown that efgartigimod is effective in gMG patients, irrespective of the disease stage or treatment history. Similarly, in this study, patients with different disease durations, clinical scales, and baseline treatment regimens demonstrated rapid improvements in MG-ADL and QMG scores after a single cycle of efgartigimod treatment. This further substantiates the conclusions drawn from the ADAPT trial. Furthermore, the use of efgartigimod did not increase the risk of infection in Chinese patients. Laboratory testing showed that the leukocytes, neutrophils, and lymphocytes remained consistently within the normal range within 8 weeks. It has been reported that efgartigimod maintained satisfactory efficacy and safety profile, even in patients experiencing exacerbations of MG triggered by infections (11).

Administration of FcRn antagonists decreases the degradation of IgG, leading to a prolonged half-life and increased concentration of serum total IgG (10, 17, 18). As an FcRn antagonist, efgartigimod effective reduced serum total IgG levels, and there is gradual rise in IgG levels after discontinuation. Moreover, we found a linear correlation between the reduction in IgG levels and the improvement in MG-ADL scores. The clearance of IgG could yield clinical benefits, and serum total IgG levels may serve as a potential biomarker for predicting the clinical efficacy of efgartigimod in gMG patients.

A retrospective study conducted in USA aimed to the integration of efgartigimod into the existing treatment algorithm for MG and assess its efficacy (19). Among the 17 patients who completed at least a 3-month treatment of efgartigimod, 15 patients (88%) reported subjective improvements in their MG-ADL scores. The mean baseline MG-ADL score was 9.1, which decreased to 3.9 at 3 months. The treatment was generally well-tolerated, with 7 patients experiencing infections. The authors recommend considering efgartigimod for patients who have failed first-line therapies, experience toxicities from their current treatments, and/or need rapid symptom improvement. Studies from Japan, Italy, and the international ADAPT+ study provide insights into the long-term efficacy and safety of efgartigimod for treating gMG across diverse populations. A multi-center study involved Japanese 1,343 gMG patients, focusing on 36 who received efgartigimod (20). Significant improvements in MG-ADL and MG-QOL15-r scores were observed. The study confirmed efgartigimod’s efficacy across various MG subtypes and concluded that efgartigimod is a feasible and well-tolerated treatment for Japanese MG patients. The Italian study (21) evaluated efgartigimod in a real-world setting and demonstrated that efgartigimod exhibited significant clinical improvements, reducing the need for hospitalization. Throughout the treatment period and a14-month follow-up, no major side effects were reported, indicating good tolerability of efgartigimod treatment. The ADAPT+ study (22), an open-label extension of the pivotal phase 3 ADAPT trial, involved 151 participants continuing efgartigimod treatment. It provided long-term data on safety, tolerability, and efficacy, showing that most participants achieved clinically meaningful improvements in MG-ADL and QMG scores over multiple treatment cycles. The study highlighted efgartigimod’s sustained efficacy and well-characterized safety profile, making it a compelling option for long-term management of gMG.

The limitations of this prospective study include the following aspects. Firstly, this study design was observational and lacked a control group, which introduced the possibility for selection or misclassification bias. Secondly, this study was limited to a single efgartigimod treatment cycle and short-term follow-up, while the ADAPT study required patients to initiate the second cycle of treatment after the change in MG-ADL scores. Extending the follow-up period would enable a comprehensive assessment of safety. Thirdly, caution should be exercised when interpreting the conclusion because of the small sample size and exclusion of MG patients with sero-negative AChR-Ab. Subsequent research could benefit from an expanded investigation using a large, multi-cycle cohort study.

## 5 Conclusion

In conclusion, this study demonstrates that efgartigimod exhibits robust efficacy and tolerability in Chinese gMG patients, with outcomes comparable to those of the ADAPT study.

## Data availability statement

The original contributions presented in the study are included in the article/Supplementary material, further inquiries can be directed to the corresponding authors.

## Ethics statement

The studies involving humans were approved by the Ethics Committee of Hainan General Hospital. The studies were conducted in accordance with the local legislation and institutional requirements. The participants provided their written informed consent to participate in this study.

## Author contributions

PW: Methodology, Data curation, Formal analysis, Validation, Writing – original draft. BZ: Data curation, Formal analysis, Methodology, Writing – original draft. JY: Formal analysis, Methodology, Validation, Writing – original draft, Investigation. JX: Formal analysis, Investigation, Methodology, Writing – original draft. YT: Formal analysis, Investigation, Methodology, Writing – original draft. FG: Formal analysis, Investigation, Methodology, Writing – original draft. FZ: Formal analysis, Investigation, Methodology, Writing – original draft. TC: Formal analysis, Investigation, Methodology, Writing – original draft. HZ: Formal analysis, Investigation, Methodology, Writing – original draft. HL: Formal analysis, Methodology, Writing – original draft, Data curation, Validation. ZZ: Data curation, Formal analysis, Methodology, Validation, Writing – original draft. HY: Validation, Conceptualization, Funding acquisition, Investigation, Project administration, Resources, Visualization, Writing – review & editing. CZ: Writing – review & editing, Conceptualization, Funding acquisition, Investigation, Methodology, Project administration, Resources, Visualization. SH: Writing – review & editing, Conceptualization, Funding acquisition, Investigation, Methodology, Project administration, Resources, Visualization.
