# Supplementary material for: Prospective cohort study evaluating efficacy and safety of efgartigimod in Chinese generalized myasthenia gravis patients
Source: Front Neurol. 2024 Jun 17;15:1407418. doi: 10.3389/fneur.2024.1407418 (PMC11222781; doi:10.3389/fneur.2024.1407418)

**Supplementary Table 1.** Clinical information and baseline data of patients

| **Patient No.** | **Age** | **Sex** | **MGFA class** | **Thymectomy** | **Prior medications** | **MG-ADL score^*^** | **QMG score^*^** | **MG-QOL15r score^*^** | **Duration since diagnosis (years)** |
| --- | --- | --- | --- | --- | --- | --- | --- | --- | --- |
| 1 | 51 | F | Ⅲa | Yes | No steroid or NSIST | 11 | 16 | 18 | 8 |
| 2 | 71 | F | IVa | Yes | Steroid and NSIST | 15 | 30 | 30 | 6 |
| 3 | 64 | M | Ⅲa | Yes | No steroid or NSIST | 12 | 23 | 23 | 11 |
| 4 | 53 | M | Ⅲa | Yes | NSIST | 9 | 14 | 12 | 1 |
| 5 | 55 | F | Ⅲa | Yes | Steroid | 17 | 19 | 23 | 5 |
| 6 | 72 | M | IVa | No | No steroid or NSIST | 11 | 23 | 19 | 0.25 |
| 7 | 34 | F | Ⅲa | No | NSIST | 7 | 21 | 10 | 15 |
| 8 | 42 | M | Ⅲa | No | NSIST | 13 | 20 | 27 | 3 |
| 9 | 66 | F | Ⅲa | No | No steroid or NSIST | 9 | 22 | 16 | 0.17 |
| 10 | 70 | F | IVa | No | NSIST | 8 | 20 | 23 | 15 |
| 11 | 66 | M | Ⅲa | No | NSIST | 9 | 14 | 16 | 0.75 |
| 12 | 71 | M | Ⅲa | Yes | Steroid and NSIST | 8 | 14 | 17 | 8 |
| 13 | 58 | F | Ⅲa | Yes | Steroid and NSIST | 7 | 16 | 16 | 6 |
| 14 | 35 | M | IVb | Yes | Steroid and NSIST | 16 | 28 | 27 | 8 |

*These assessments were conducted at baseline (3 days before efgartigimod administration). F, female. M, male. NSIST, non-steroidal immunosuppressants. MG-ADL, myasthenia gravis activities of daily living. QMG, quantitative myasthenia gravis. MG-QOL 15r, 15-item Myasthenia Gravis Quality of Life Scale. MGFA, myasthenia gravis foundation of America.

**Supplementary Figure 1.** Schematic design of the NPP.

**
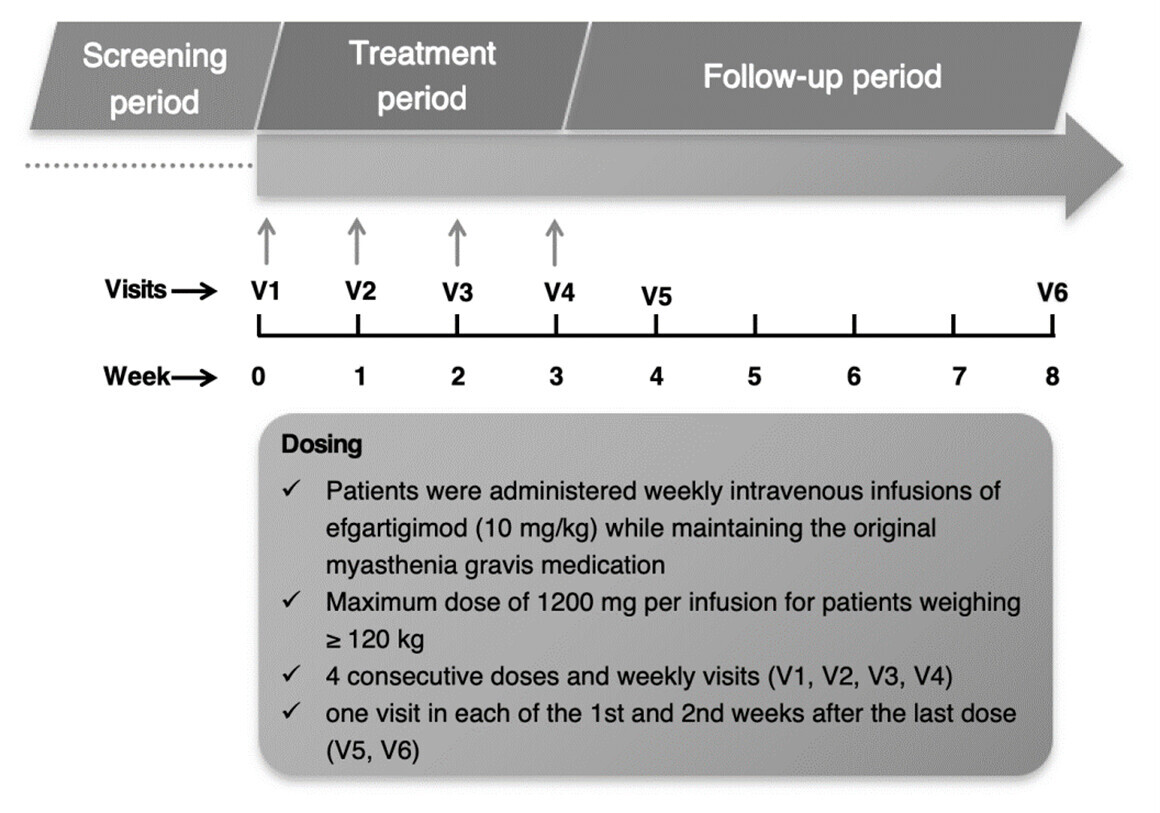
**

**Supplementary Figure 2.** Changes in laboratory parameters between the treatment and follow-up periods.


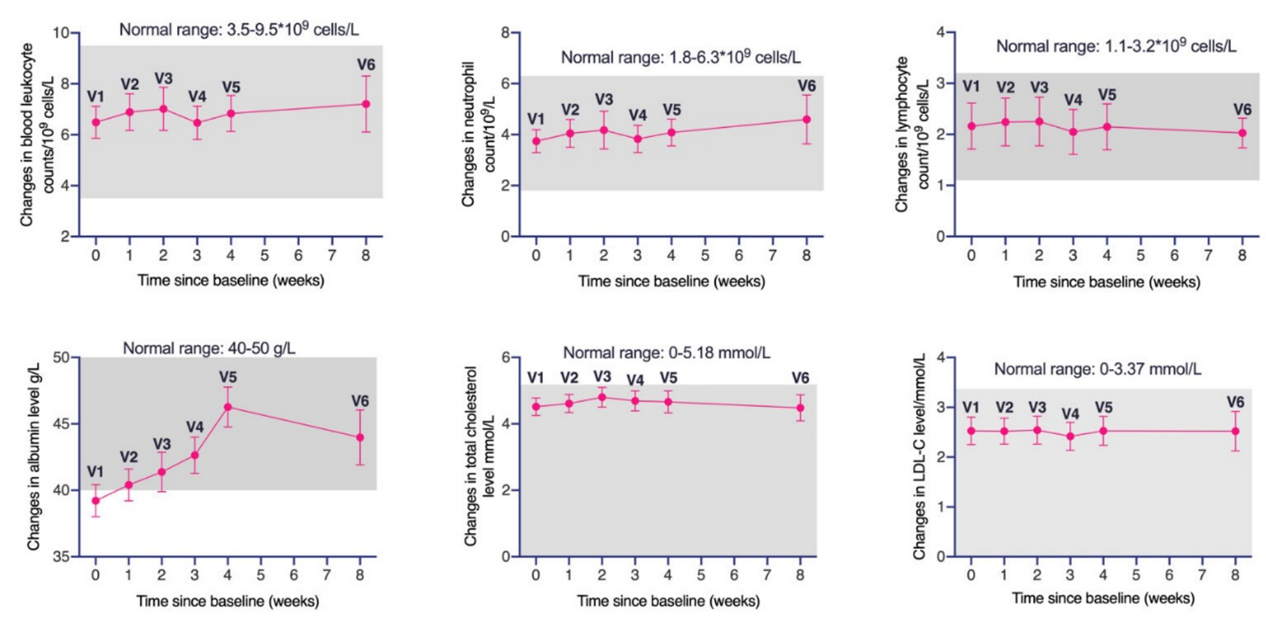


**Supplementary Figure 3.** Changes in the average IgG level in between the treatment and follow-up periods.


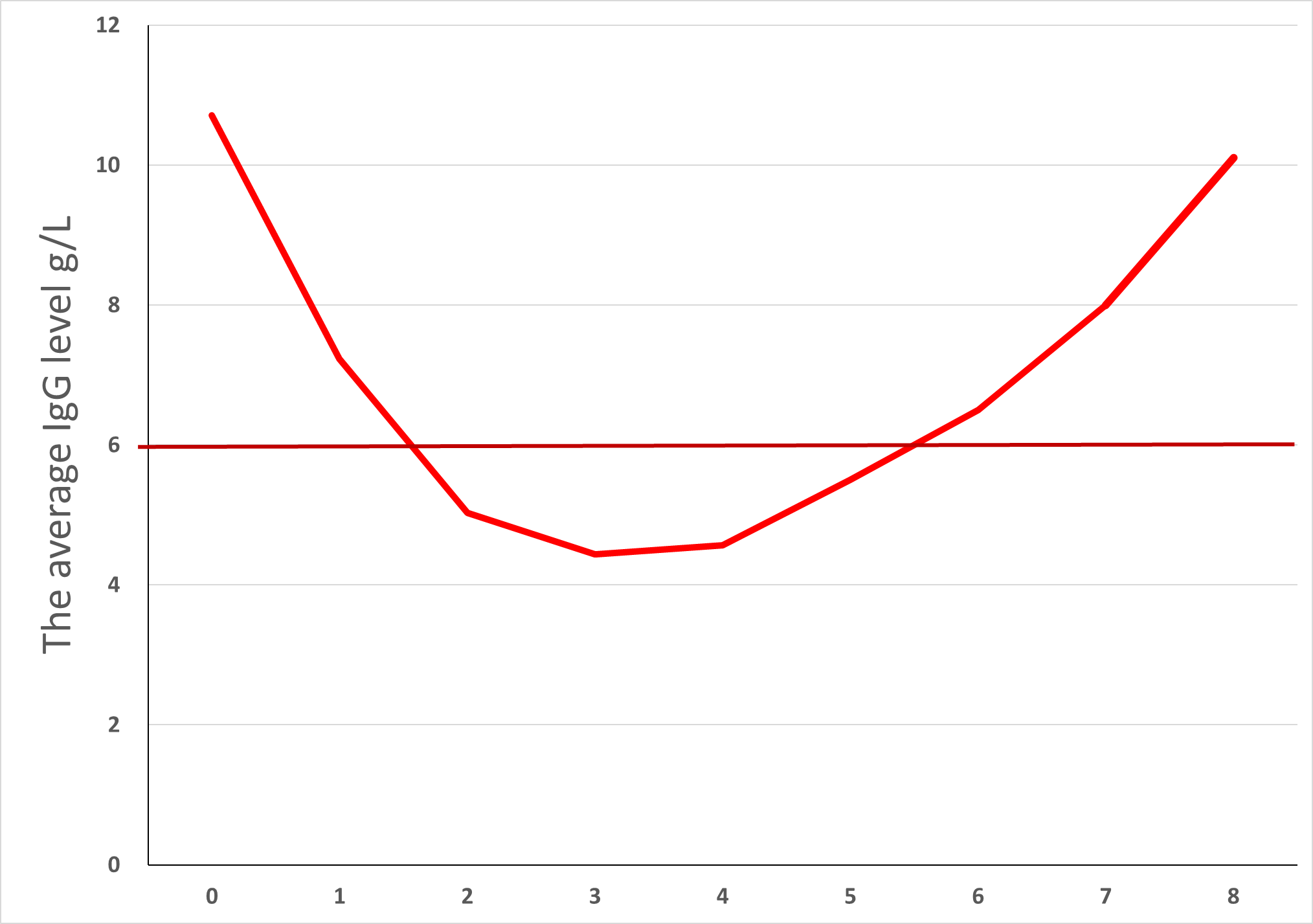

Supplement: Supplementary file 1 [file Data_Sheet_1.docx]
